# Papillary thyroid neoplasm: An incidental malignant nodule

**DOI:** 10.1002/ccr3.7364

**Published:** 2023-05-16

**Authors:** Mahfujul Z. Haque, Michael Burcescu, Zirak Sajjad

**Affiliations:** ^1^ Michigan State University College of Human Medicine Grand Rapids Michigan USA; ^2^ Detroit Medical Center Detroit Michigan USA

**Keywords:** endocrine cancer, neoplasm, papillary thyroid cancer, thyroid nodule

## Abstract

Papillary thyroid cancer (PTC), a common neoplasm originating from thyroid follicular cells, represents 85% of thyroid malignancy. PTC is known to metastasize to adjacent structures. Studies report that 5–15% of detected thyroid nodules represent malignancy; here, we report a 51‐year‐old woman with incidental thyroid nodules identified on the cervical spine.

## Graphical abstract

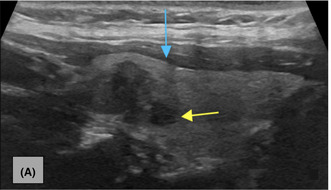

The patient is a 51‐year‐old African American woman with incidental thyroid nodules identified on MRI of the cervical spine. Patient describes a tender palpable right thyroid nodule but is otherwise asymptomatic. Patient denies a family history of thyroid cancer. There is no history of tobacco use or toxic occupational exposure. There is no palpable neck mass. Thyroid panel was within normal limits.

Figure 1 consists an MRI series of the cervical spine demonstrating the thyroid nodule with axial‐T1, traditional‐T1, and T2 views. Figure 2 consists of a longitudinal‐axis ultrasound and transverse ultrasound that reveal a right thyroid lobe with a 1.5 × 1.0 × 1.3 cm hypoechoic solid nodule with irregular margins, punctate echogenic foci, and mildly increased vascularity. Figure 3 demonstrates a longitudinal‐axis ultrasound with Doppler investigation of the right thyroid lobe with a slight enlargement of the nodule, measuring 1.5 × 1.2 × 1.3 cm, with hypoechoic wider than tall features, and heightened blood flow to the nodule**.** Figure 4 longitudinal‐axis ultrasound demonstrates further nodule enlargement to 1.9 × 1.3 × 0.9 cm. These findings of irregular margins, and punctate echogenic foci, and vascular changes, are consistent with a TI‐RADS 5 nodule and suggestive of malignancy.

**FIGURE 1:**
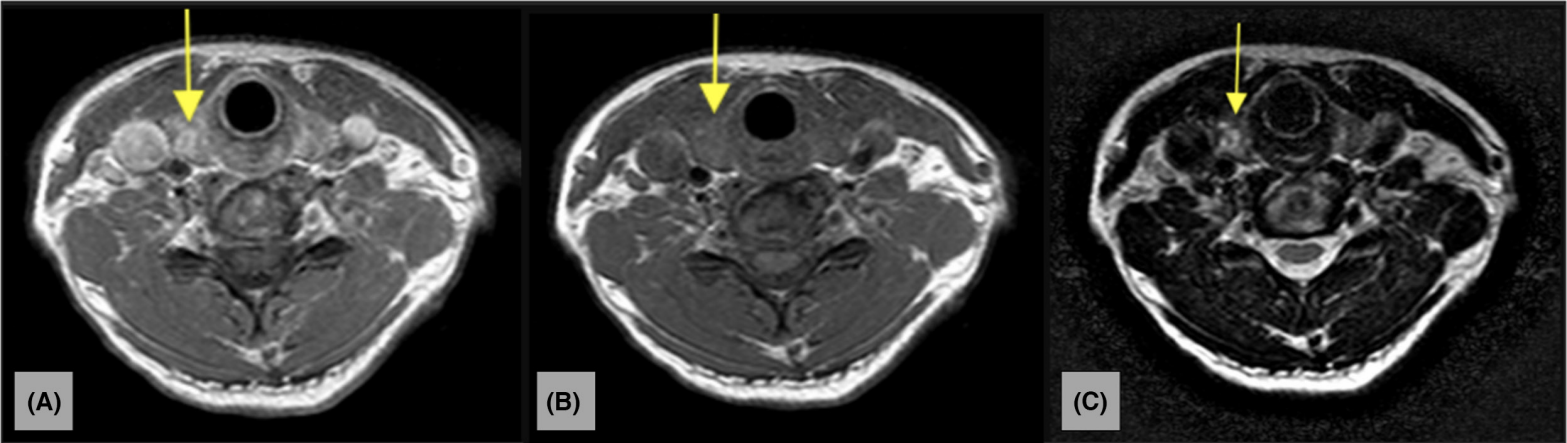
(A) Axial T1‐weighted image, after IV gadolinium administration, of the cervical spine demonstrates an incidental enhancing right thyroid lobe nodule (yellow arrow). (B) Axial T1‐weighted image through the cervical spine demonstrates T1 isointense nodularity of the right thyroid lobe (yellow arrow). (C) Axial T2‐weighted image through the cervical spine demonstrates T2 hyperintense nodularity of the right thyroid lobe (yellow arrow). Keywords: ACR TI‐RADS Criteria.

**FIGURE 2:**
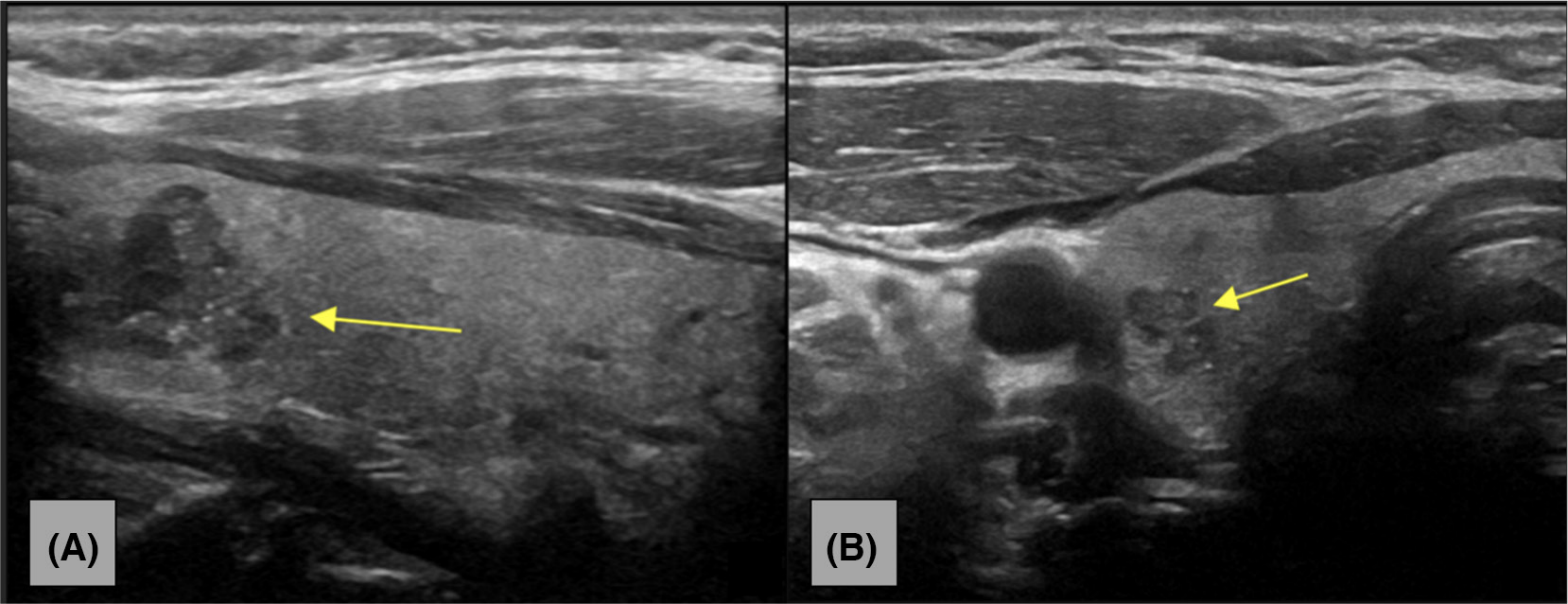
(A) Long‐axis ultrasound image of the right thyroid lobe demonstrates a 1.5 × 1.0 × 1.3 cm hypoechoic wider than tall solid nodule with irregular margins and punctate echogenic foci (yellow arrow). Right thyroid lobe ultrasound in longitudinal (A, B) and transverse (C, D, E) planes demonstrate a 1.5 × 1.0 × 1.3 cm hypoechoic wider than tall solid nodule with irregular margins and punctate echogenic foci (yellow arrow). Mildly increased vascularity is exhibited on color Doppler investigation (B, D). (B) Transverse ultrasound image of the right thyroid lobe demonstrates a 1.5 × 1.0 × 1.3 cm hypoechoic wider than tall solid nodule with irregular margins and punctate echogenic foci (yellow arrow). Keywords: ACR TI‐RADS Criteria.

**FIGURE 3:**
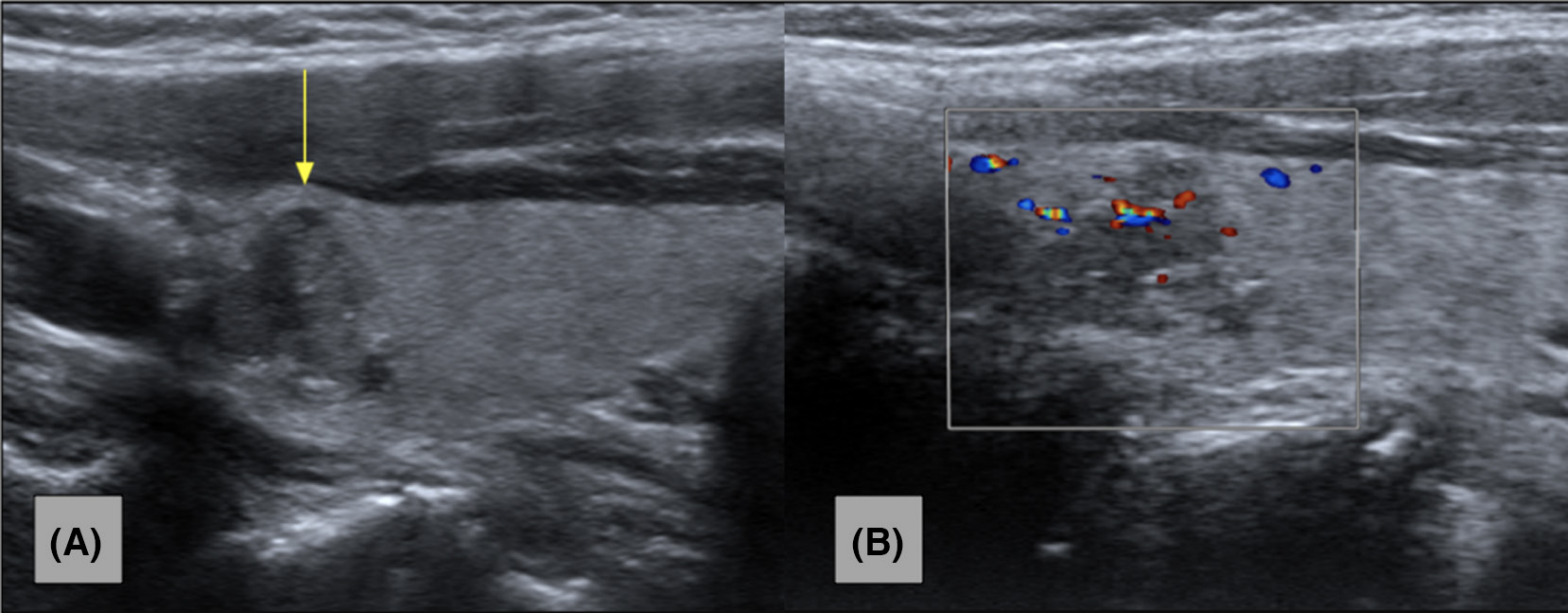
(A) Longitudinal‐axis ultrasound image of the right thyroid lobe demonstrates slight nodule enlargement to 1.5 × 1.2 × 1.3 cm (yellow arrow). Hypoechoic wider than tall features with irregular margins and punctate echogenic foci appear similar to previous sonographic evaluation. (B) Longitudinal‐axis color Doppler image of the right thyroid lobe demonstrates increased vascularity of the thyroid nodule. Keywords: ACR TI‐RADS Criteria.

**FIGURE 4:**
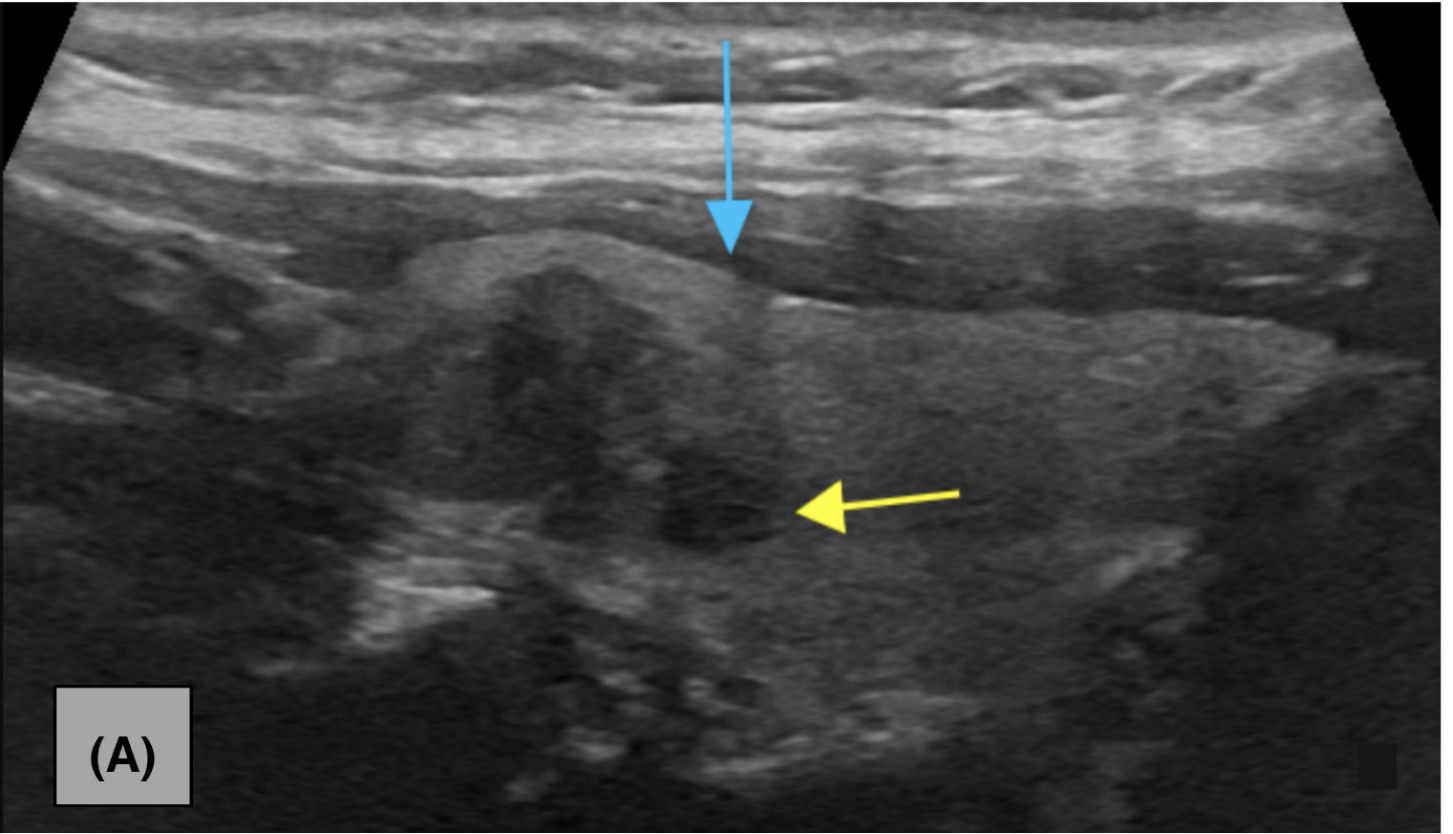
(A) Longitudinal‐axis ultrasound image of the right thyroid lobe demonstrates further nodule enlargement to 1.9 × 1.3 × 0.9 cm. There is a very hypoechoic echotexture of the nodule inferior aspect (yellow arrow) when compared to overlying musculature (blue arrow).

A diagnosis of parathyroid adenoma was considered. However, parathyroid adenomas are located adjacent and posterior or just inferior to the thyroid gland. Rarely, the parathyroid gland may mimic thyroid tissue by becoming adenomatous and demonstrate a peripheral rim of hyper‐vascularity. The irregular margins and punctate echogenic foci demonstrated within this nodule are more consistent with a TI‐RADS 5 nodule.

Fine needle aspiration biopsy of right upper thyroid nodule showed moderately cellular with cohesive groups as sheets and singly scattered cells exhibiting nuclear grooves, powdery chromatin, and irregular nuclear outlines. A few intranuclear pseudo‐inclusions are seen with some colloids in the background. True papillae, psammoma bodies, and necrosis were not detectable despite sampling with three adequate passes.

The patient's findings were most consistent with a papillary thyroid neoplasm. The patient was scheduled for surgical resection with follow‐up sonography and chemistry panels 1‐year after resection. The patient was lost to follow‐up before being aware of the final pathology results.

Studies report that 5–15% of all detected thyroid nodules and up to 11% of incidental thyroid nodules represent malignancy.^2^ The SEER database reports the USA incidence of thyroid carcinoma to be 14.9 per 100,000 with a 1:2.8 male‐to‐female predilection.^3^ Papillary thyroid cancer (PTC) is invasive and known to metastasize to adjacent structures. Established risk factors for thyroid cancer include radiation exposure, family history of thyroid cancer, occupational exposure, and obesity.^1^

PTC is associated with favorable mortality of 11–17% and a low recurrence rate of 5–15%.^1^ The primary treatment for PTC is surgical. Preprocedural considerations include tumor size, metastases, extra‐thyroidal extension, and airway compromise. Sonographic and biochemical recurrence monitoring is typically performed at 6–12‐month intervals for at least 5 years.

In conclusion, this case highlights the importance of thorough evaluation and monitoring of incidental thyroid nodules, as they may represent malignancy.

## AUTHOR CONTRIBUTIONS

**Mahfujul Z. Haque:** Project administration; validation; visualization; writing – original draft; writing – review and editing. **Michael Burcescu:** Conceptualization; data curation; formal analysis; investigation; project administration; supervision. **Zirak Sajjad:** Conceptualization; validation; visualization; writing – original draft; writing – review and editing.

## FUNDING INFORMATION

None.

## CONFLICT OF INTEREST STATEMENT

None.

## CONSENT

Written informed consent was obtained from the patient to publish this report in accordance with the journal's patient consent policy.

## Data Availability

The data that support the findings of this study are available on request from the corresponding author. The data are not publicly available due to privacy or ethical restrictions.
